# Protective Effect of Resveratrol on Immortalized Duck Intestinal Epithelial Cells Exposed to H_2_O_2_

**DOI:** 10.3390/molecules27113542

**Published:** 2022-05-31

**Authors:** Ning Zhou, Yong Tian, Hongzhi Wu, Yongqing Cao, Ruiqing Li, Kang Zou, Wenwu Xu, Lizhi Lu

**Affiliations:** 1 State Key Laboratory for Managing Biotic and Chemical Threats to the Quality and Safety of Agro-Products, Institute of Animal Science & Veterinary, Zhejiang Academy of Agricultural Sciences, Hangzhou 310000, China; 2017205001@njau.edu.cn (N.Z.); tyong@zaas.ac.cn (Y.T.); 2017805096@njau.edu.cn (Y.C.); lrqgy0601@outlook.com (R.L.); 2 College of Animal Science and Technology, Nanjing Agricultural University, Nanjing 210095, China; kangzou@njau.edu.cn; 3 Tropical Crop Genetic Resource Research Institute, Chinese Academy of Tropical Agricultural Sciences, Haikou 571101, China; hong-zhi@catas.cn

**Keywords:** resveratrol, epithelial cells, duck, oxidative stress, apoptosis

## Abstract

Resveratrol is a polyphenolic compound with anti-oxidation effects. The mechanisms underlying the antioxidant effects of resveratrol in duck intestinal epithelial cells remain unclear. The protective effects of resveratrol against oxidative stress induced by H_2_O_2_ on immortalized duck intestinal epithelial cells (IDECs) were investigated. IDECs were established by transferring the lentivirus-mediated simian virus 40 large T (*SV40T*) gene into small intestinal epithelial cells derived from duck embryos. IDECs were morphologically indistinguishable from the primary intestinal epithelial cells. The marker protein cytokeratin 18 (CK18) was also detected in the cultured cells. We found that resveratrol significantly increased the cell viability and activity of catalase and decreased the level of intracellular reactive oxygen species and malondialdehyde, as well as the apoptosis rate induced by H_2_O_2_ (*p* < 0.05). Resveratrol up-regulated the expression of NRF2, p-NRF2, p-AKT, and p-P38 proteins and decreased the levels of cleaved caspase-3 and cleaved caspase-9 and the ratio of Bax to Bcl-2 in H_2_O_2_-induced IDECs (*p* < 0.05). Our findings revealed that resveratrol might alleviate oxidative stress by the PI3K/AKT and P38 MAPK signal pathways and inhibit apoptosis by altering the levels of cleaved caspase-3, cleaved caspase-9, Bax, and Bcl-2 in IDECs exposed to H_2_O_2_.

## 1. Introduction

Modern large-scale intensive farming operations are high-stress environments for ducks and can substantially affect their intestinal health. The intestinal epithelium consists of intestinal epithelial cells, which lie at the interface between the internal environment and the intestinal lumen and mediate the homeostatic regulation of the intestinal barrier [1]. The intestinal epithelial cells not only act as a physical barrier but also play a key role in preventing macromolecules and pathogenic microorganisms from penetrating the mucosa and inducing mucosal oxidative stress and a systemic inflammatory response [2,3]. In vitro systems involving cultured intestinal epithelial cells are suitable models for studying oxidative stress and the inflammatory response. Primary intestinal epithelial cells possess a finite life span owing to replicative senescence, precluding their long-term culture [4]. Immortalized cell lines can maintain several characteristics following numerous passages, which not only provides a consistent supply of cells but also increases the reproducibility of experimental results [5]. A stable and reliable intestinal epithelial cell line is needed to facilitate research on oxidative stress mechanisms and the effects of antioxidants and treatments.

There are two main methods for establishing a cell line: (1) transfection of vectors that can express the human telomerase reverse transcriptase (*hTERT*) gene [6,7,8]; (2) transfection of a plasmid containing the SV40 large T antigen (*SV40T*) gene [9,10]. SV40 is a eukaryotic cell virus that contains the large T antigen and small T antigen; the former is a dominant-acting protein that can modify the regulation of the cell cycle of host cells and immortalize primary cells [11]. *SV40T* has a higher transfection and expression efficiency compared with *hTERT* [10].

Oxidative stress, which refers to cellular or molecular damage caused by reactive oxygen species (ROS), stems from the disruption of the balance between ROS production and the antioxidant defense response [12]. Excess ROS alters the levels of malondialdehyde (MDA), catalase (CAT), glutathione peroxidase (GSH-PX), and superoxide dismutase (SOD) and damages nucleic acids, proteins, and lipids in cells, which leads to cell apoptosis [13]. The transcription factor NRF2 (nuclear factor erythroid 2-related factor 2) is a highly conserved protective molecule that regulates the response to oxidative stress in intestinal epithelial cells [14]. Because of the hazards posed by oxidative stress in the intestine, antioxidants that can effectively decrease ROS production and scavenge free radicals may promote the increased apoptosis of epithelial cells.

Resveratrol, which is a natural plant polyphenol from several plants such as grapes, blueberries, and mulberries, is thought to have antioxidant and anti-inflammatory effects [15]. Several studies have demonstrated that resveratrol acts on multiple cellular proteins such as NRF2, AKT, and P38 to affect processes related to oxidative stress and apoptosis reduction [16,17,18]; in addition, resveratrol can improve the antioxidative status of muscle [19] and regulate the serum metabolic parameters of broilers under heat stress [20]. However, few studies have examined the protective effect of resveratrol on duck intestinal epithelial cells, and the molecular mechanisms underlying its antioxidant activity remain unclear. Hydrogen peroxide (H_2_O_2_) is a strong oxidizing agent that has often been used to establish oxidative stress models [18,21]. Here, we explored the effects of resveratrol on the proliferation of immortalized duck intestinal epithelial cells (IDECs) and the mechanism of cell apoptosis under oxidative stress induced by H_2_O_2_. Our results provide new insights that could be used to aid future applications of resveratrol as feed additives to mitigate intestinal damage in livestock.

## 2. Material and Methods

### 2.1. Isolation and Culture of Primary Duck Intestinal Epithelial Cells

Isolation and culture of cells were performed following previously published methods [22]. Healthy 20-d duck embryos (Shaoxing Duck) from eggs with shells that have started to break were used in experiments. After the abdominal cavity was opened, the small intestines were isolated in a sterile environment and transferred to a 90-mm Petri dish (Corning, NY, USA) filled with warm (37 °C) phosphate-buffered saline (PBS, Solarbio, Beijing, China) containing 2% streptomycin/penicillin (Gibco, NY, USA). The mesentery and mesenteric blood vessels were carefully removed and discarded. The lumen was rinsed with PBS by a syringe twice and cut open with ophthalmic scissors; the tissue was then minced into several pieces (approximately 1 mm^3^) and rinsed three times with PBS. Tissue blocks were digested with Dulbecco’s Modified Eagle’s Medium/F12 (DMEM/F12) (Gibco, NY, USA) supplemented with type I collagenase (Sigma, St. Louis, MO, USA) for 70 min at 37 °C on a shaking table. After centrifugation (500× *g*, 4 min), the cells were collected, and the supernatant was discarded. The cells were then resuspended in complete medium and seeded into a T25 cell culture flask (Corning, NY, USA). After 70 min, non-adherent cells were collected and seeded into a new T25 cell culture flask, and this process was repeated three times. The cells were incubated at 37 °C in an incubator with 5% CO_2_, and the medium was refreshed every two days. The complete medium contained DMEM/F12, 5% fetal bovine serum (Gibco, NY, USA), 1% streptomycin/penicillin (Gibco, NY, USA), 1% insulin-transferrin-selenium (Invitrogen, Carlsbad, CA, USA), 1% N-2-hydroxyethylpiperazine-N-2-ethane sulfonic acid (Solarbio, Beijing, China), and 10 ng/mL EGF (Sigma, St. Louis, MO, USA).

### 2.2. Immortalization of Primary Duck Intestinal Epithelial Cells Using Lentiviral Vectors

Human 293T cells were used for lentiviral packaging. The lentiviral vector pLenti *SV40T* purchased from Applied Biological Materials (Abm, Vancouver, BC, Canada) Inc., lentiviral package vector 2nd Generation Packaging Mix (Abm, Vancouver, BC, Canada), and Lentifectin™ Transfection Reagent (Abm, Vancouver, BC, Canada) were co-transfected into human 293T cells to produce lentiviral particles. The medium was collected at 48 h and filtered with a 0.45-μm filter (Millipore, Billerica, MA, USA). The viral supernatants were mixed with 60% 5 × PEG8000 and centrifuged at 10,000× *g* for 4 h. After discarding the supernatant, sedimentary lentiviral particles were resuspended in DMEM/F12 medium, and the viral titer was determined by a Lentivirus Titer Kit (Abm, Vancouver, BC, Canada). Primary duck intestinal epithelial cells were infected with lentivirus at a multiplicity of infection of 10 and cultured at 37 °C for 48 h; the clonal population was selected by the ability of the immortalized cells confirmed by continuous culture past 20 passages to survive senescence [23]. Immortalized cells were confirmed by continuous culture past 30 passages and subsequently referred to as IDECs.

### 2.3. RNA Extraction and Quantitative Polymerase Chain Reaction (qPCR)

RNA was extracted from primary duck intestinal epithelial cells and immortalized cells using an ExCellenCT Lysis Kit (Abm, Vancouver, BC, Canada) and reverse-transcribed into cDNA using 5X All-In-One RT MasterMix (Abm, Vancouver, BC, Canada) per the manufacturer’s protocol. The quality and quantity of RNA were measured using a Nanodrop (Thermo Fisher, Waltham, MA, USA) at 260 nm and 280 nm, respectively. Real-time PCR was performed using EvaGreen 2X qPCR MasterMix (Abm, Vancouver, BC, Canada) with an ABI PCR detection system. The following conditions were used for the qRT-PCR reaction: 95 °C for 1 min; 40 cycles of 95 °C for 10 s, 60 °C for 5 s, and 72 °C for 15 s; and a final extension of 72 °C for 10 min. The primer pairs used for detection are listed in Table 1.

### 2.4. Immunofluorescence Microscopy and Cell Growth Curves

Cells were seeded into 6-well plates (Corning, NY, USA) and fixed with 4% paraformaldehyde (Solarbio, Beijing, China) for 20–30 min. After washing three times with PBS for 5 min each, cells were permeabilized with 0.1% Triton X-100 for 20 min at room temperature and blocked with 3% bovine serum albumin (Servicebio, Wuhan, China) for 30 min at room temperature. Cells were then immunolabeled with anti-cytokeratin 18 antibodies (1:500 mouse monoclonal, Abcam, Cambridge, MA, USA) overnight at 4 °C. Moreover, samples were washed with PBS for 5 min three times and incubated with monkey anti-mouse IgG (H+L) secondary antibody (Proteintech, Chicago, IL, USA) at room temperature for 50 min. After washing three times in PBS, cells were DAPI (Servicebio, Wuhan, China) stained for 10 min at room temperature in the dark and imaged under a fluorescent inverted microscope (Thermo Fisher, Waltham, MA, USA). The method of cell growth curves was based on a previous article [8]. Primary duck intestinal epithelial cells (P7) and IDECs (P30) were plated onto 24-well plates (Corning, NY, USA) at 2.5 × 10^4^ cells per well and incubated at 37 °C with 5% CO_2_. One well of cells was calculated by a red blood cell counting board (Qiujing, Shanghai, China) every 24 h. Experiments were conducted in triplicate, and mean values were plotted for the growth curves.

### 2.5. Cell Treatments

After IDECs were treated with 100 μM, 200 μM, 300 μM, 400 μM, and 500 μM H_2_O_2_ (Sigma, St. Louis, MO, USA) for 2 and 6 h, H_2_O_2_ was diluted in dimethyl sulfoxide (DMSO, Sigma, St. Louis, MO, USA). The cells were pretreated with a series of different concentrations of resveratrol (0, 5, 10, 25, and 50 μM, Selleck, Houston, TX, USA) for 6 h before incubating with H_2_O_2_ [18]; the cells were then collected for further study.

### 2.6. Viability of IDECs

IDECs were treated under different conditions, and cell viability was tested using the CCK8 assay. Briefly, cells (1.2 × 10^4^ /well) were seeded in 96-well plates and cultured until the confluence reached approximately 80%. Cell viability was detected using a CCK-8 assay kit (Dojindo, Kumamoto, Japan) after treatment with different concentrations of H_2_O_2_ for different lengths of time. The absorbance at 450 nm of each group was measured by a PerkinElmer Envision plate reader (Thermo Fisher, Waltham, MA, USA).

### 2.7. Antioxidant Indices and ROS Level

IDECs were plated at 5 × 10^6^ cells/well in a six-well plate overnight and treated under different conditions. The cells were then harvested and disrupted ultrasonically on ice, and the activity of MDA, CAT and GSH-PX was determined using commercial assay kits (Jiancheng, Nanjing, China) per the manufacturer’s instructions. Data were normalized to the protein concentration, which was determined by a bicinchoninic acid (BCA) protein assay kit (Beyotime, Shanghai, China). The relative levels of intracellular ROS were measured using a commercial ROS detection kit (Solarbio, Beijing, China). Cells were washed twice with PBS and then incubated with 2′,7′-dichlorofluorescein diacetate (DCFH-DA) for 20 min at 37 °C. The fluorescence intensity was measured by a PerkinElmer Envision plate reader (Thermo Fisher, Waltham, MA, USA).

### 2.8. Flow Cytometry Analysis of Apoptosis

Cell apoptosis analysis was performed by flow cytometry (CytoFLEX S, Beckman, Pasadena, CA, USA). Following each specific treatment, cells were collected, washed twice with ice-cold PBS, and then centrifuged at 500× *g* for 5 min. Cells were resuspended in Annevix Binding Buffer from an Apoptosis Detection Kit (Vazyme, Nanjing, China), and samples were transferred to sterile flow cytometry glass tubes. Cells were then incubated with Annevix FITC and PI at room temperature under dark conditions for 15 min. Flow cytometric analysis was conducted per the manufacturer’s instructions.

### 2.9. Western Blot Analysis

The treated IDECs were lysed in RIPA buffer (Beyotime, Shanghai, China) containing 1 mmol/L PMSF (Beyotime, Shanghai, China) on ice and centrifuged at 12,000 rpm at 4 °C for 10 min. Total proteins were further quantified using a BCA kit (Beyotime, Shanghai, China). After samples were mixed with a fifth of the SDS loading buffer (Pujian, Wuhan, China) and boiled at 100 °C for 5 min, samples were separated by sodium dodecyl sulfate-polyacrylamide gel electrophoresis (SDS-PAGE, Solarbio, Beijing, China), transferred to a PVDF membrane (Pall, NY, USA), and then incubated with 5% skim milk for 1 h at room temperature to block the non-specific binding proteins. Next, the membranes were incubated with primary antibodies, including P38 (1:1000, Proteintech, Chicago, IL, USA), p-P38 (Thr180/tyr182, 9211S) (1:1000, CST, Danvers, MA, USA), AKT (1:1000, Proteintech, Chicago, IL, USA), p-AKT (Thr308, 9275S) (1:1000, CST, Danvers, MA, USA), NRF2 (1:500, Bioss, Beijing, China), p-NRF2 (Ser40, BS-2013R) (1:500, Bioss, Beijing, China), Bax (1:1000, Proteintech, Chicago, IL, USA), Bcl-2 (1:1000, Proteintech, IL, Chicago, USA), caspase-3 (1:1000, Proteintech, Chicago, IL, USA), and caspase-9 (1:1000, Proteintech, IL, Chicago, USA), and GAPDH (1:5000, Pujian, Wuhan, China) overnight at 4 °C and then were incubated with secondary antibody (1:5000, Chicago, Proteintech, USA) at room temperature for 2 h. Finally, the membranes were visualized with enhanced chemiluminescence (ECL, Biosharp, Chengdu, China) reagent and analyzed by ImageJ software. All protein measurements were normalized to GAPDH.

### 2.10. Statistical Analysis

Data were analyzed by one-way analysis of variance (ANOVA), followed by a least significant difference (LSD) post hoc test and two-sided Student’s *t*-test. The data were expressed as the mean ± standard error (SE) and plotted using GraphPad Prism 8.3 software. Statistical analyses were performed using SPSS 15.0. The threshold for statistical significance was *p* < 0.05.

## 3. Results

### 3.1. Primary Cells Morphology

**Figure 1:**
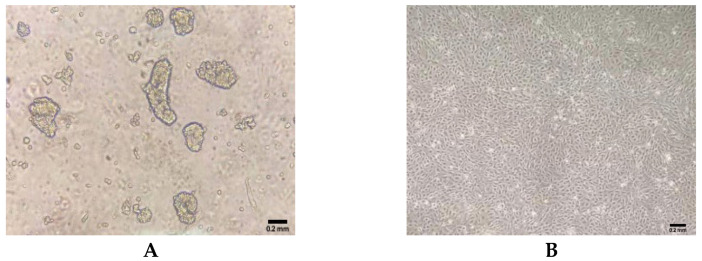
Morphology of primary duck intestinal epithelial cells isolated from duck embryos. (**A**) Cellular morphology in 6 h in culture after isolation. (**B**) Cellular morphology at passage 3 in culture.

The primary intestinal epithelial cells of ducks were isolated by enzymatic digestion, and the morphology of primary cells was observed under an inverted microscope. Within 6 h after the initial culture, single cells and cell clusters began to adhere to the culture flask (Figure 1A). During the third passage, epithelial cell sheets exhibited a homogeneous cobblestone-like morphology (Figure 1B).

### 3.2. Establishment of IDECs

**Figure 2:**
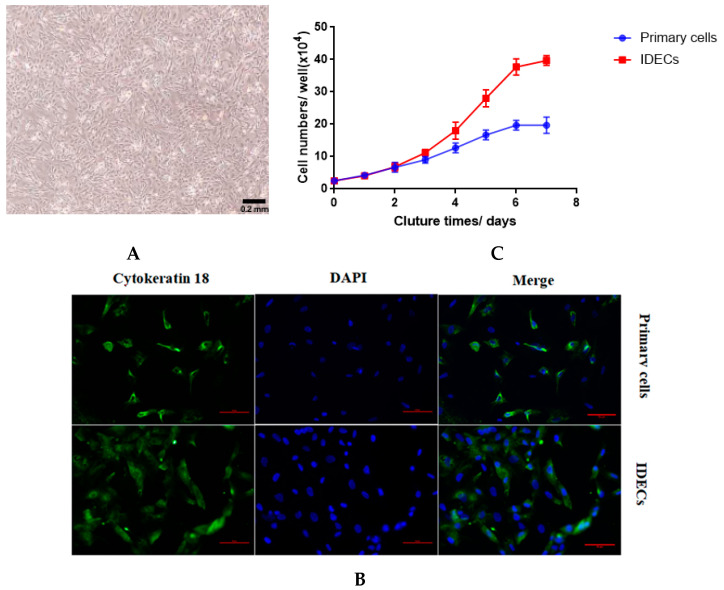
Characterization of immortalized duck intestinal epithelial cells(IDECs). (**A**) Morphology of IDECs at passage 30 under microscope. (**B**) The immunofluorescence study for cytokeratin 18. (**C**) Primary cells and IDECs were cultured for 7 days and average cell numbers were counted on different days. Data are represented as the mean ± SE (*n* = 3).

To establish IDECs, we transfected *SV40T* into the primary duck intestinal epithelial cells at passage three from an E20 duck. Similar to primary epithelial cells, IDECs grew in a monolayer and possessed a typical cobblestone-like morphology (Figure 2A). To determine whether *SV40T* was transferred into primary duck intestinal epithelial cells, we used qRT-PCR to detect the expression of the *SV40T* gene in primary duck intestinal epithelial cells and IDECs. *SV40T* mRNA was detected in IDECs but was not detected in primary cells (Table 2), suggesting that the *SV40T* gene was successfully integrated and expressed in IDECs.

The biological attributes of the primary cells and IDECs were then evaluated using molecular markers. Immunofluorescence with anti-cytokeratin 18 antibodies revealed that cytokeratin 18 (CK18) was present in primary cells and IDECs (Figure 2B). To assess the proliferation characteristics of IDECs, growth curves of IDECs were created and compared with those of primary cells. The proliferation rate of IDECs was higher than that of primary cells (Figure 2C).

### 3.3. Effect of H_2_O_2_ on the Antioxidant Ability and Apoptosis of IDECs

**Figure 3:**
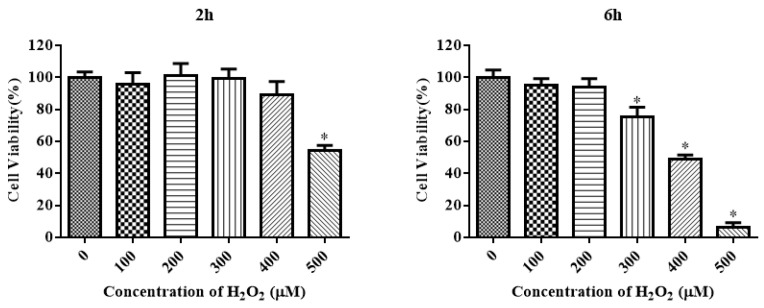
The IDECs were treated with various concentrations and incubation times of H_2_O_2_ (0–500 µM, 2 h and 6 h) and the cell viability was estimated by CCK-8 assay. The data were presented as percent viability of IDECs when treated with different conditions of H_2_O_2_. The data were analyzed through one-way ANOVA and are expressed as the mean ± SE (*n* = 3). * *p* < 0.05 compared to 0 µM H_2_O_2_.

The CCK-8 assay method was used to measure the viability of IDECs to aid the selection of appropriate concentrations of H_2_O_2_ with different treatment times to induce oxidative stress. H_2_O_2_ reduced the cell viability in a time-dependent and dose-dependent manner (Figure 3). The viability of IDECs decreased by 57.36% (*p* < 0.05) after 500 μM H_2_O_2_ treatment for 2 h, whereas 400 μM H_2_O_2_ treatment for 6 h decreased cell viability to 48.89% (*p* < 0.05) compared with the control. Based on these results, we conducted further experiments using the 200 μM and 400 μM H_2_O_2_ treatments for 6 h.

**Figure 4:**
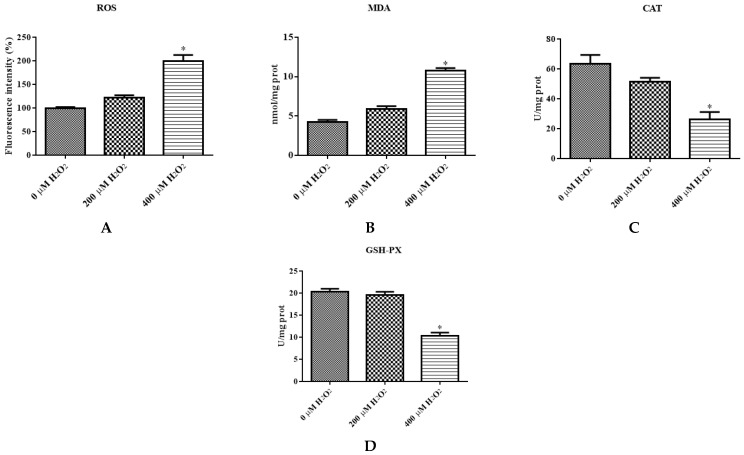

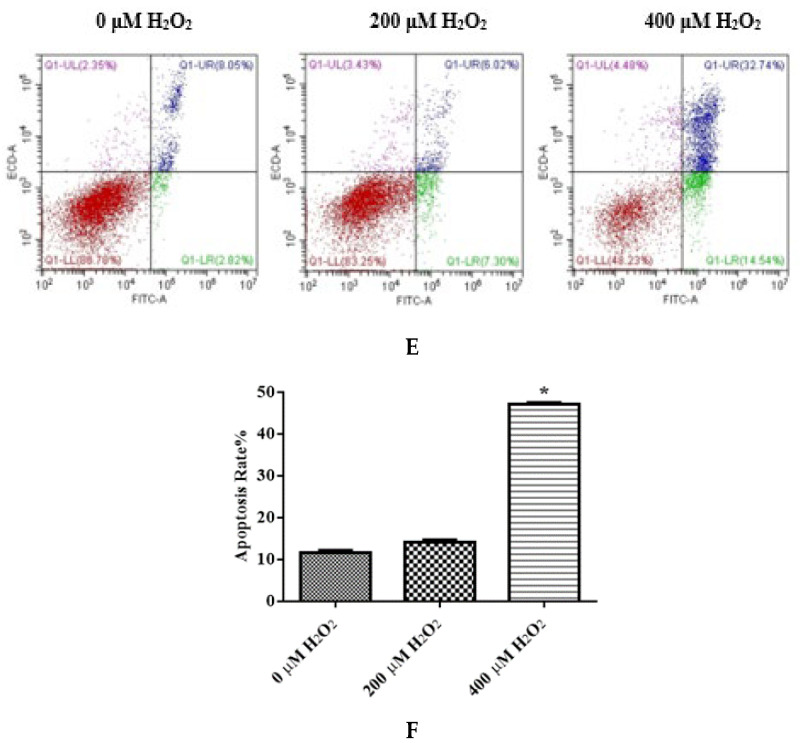
The IDECs were treated with 0 µM H_2_O_2_, 200 µM H_2_O_2_ and 400 µM H_2_O_2_ for 6 h. (**A**) The ROS production in IDECs treated with different strategies was determined by 2′,7′-dichlorofluorescein diacetate (DCFH-DA). (**B**) The activities of MDA in IDECs were detected by commercial kits. (**C**) The activities of CAT in IDECs were detected by commercial kits. (**D**) The activities of GSH-PX in IDECs were detected by commercial kits. (**E**) Apoptosis cells were analyzed by flow cytometry using Apoptosis Detection Kit. (**F**) The data were analyzed through one-way ANOVA and are expressed as the mean ± SE (*n* = 3). * *p* < 0.05 compared to 0 µM H_2_O_2_.

The levels of different ROS, MDA, CAT, and GSH-PX of IDECs were detected by a PerkinElmer Envision plate reader. The ROS level was significantly increased in the 400 μM H_2_O_2_ treatment (*p* < 0.05) compared with the negative control (Figure 4A). The MDA content of cells increased dramatically after 400 μM H_2_O_2_ treatment compared with the negative control (*p* < 0.05), and the activity of CAT and GSH-PX was significantly decreased in the 400 μM H_2_O_2_ treatment group compared with the control group (*p* < 0.05) (Figure 4B–D).

To determine whether apoptosis is associated with reduced cell viability, cells were stained with Annexin V/PI to determine the apoptosis rate. The total apoptosis rate of IDECs was markedly increased in the 400 μM H_2_O_2_ treatment (*p* < 0.05) (Figure 4E,F). Subsequent experiments of IDECs were conducted with 400 μM H_2_O_2_ for 6 h.

### 3.4. Effect of Resveratrol on the Viability of IDECs

**Figure 5:**
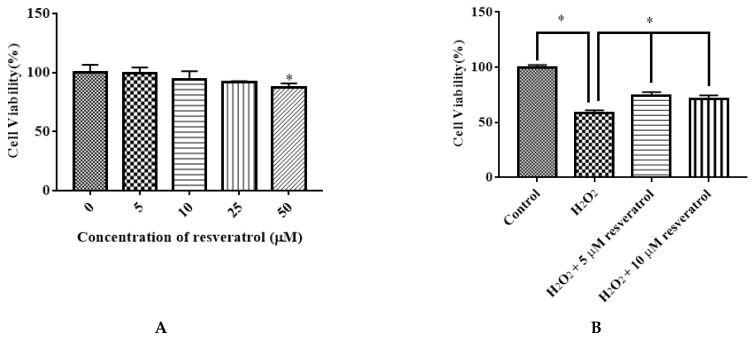
Effects of resveratrol on the viability of IDECs. (**A**) IDECs cells were incubated with increasing concentrations of resveratrol for 6 h. * *p* < 0.05 compared to 0 μM resveratrol. (**B**) IDECs were pretreated with the resveratrol (5 µM and 10 µM) and then cocultured with 400 μM H_2_O_2_ for 6 h. Then cell viability was measured by CCK-8 assays. The data were analyzed through one-way ANOVA and are expressed as the mean ± SE (*n* = 3). * *p* < 0.05 compared to H_2_O_2_ group.

We examined the effect of different doses of resveratrol (5, 10, 25, and 50 μM) on the viability of IDECs. Resveratrol had little effect on cell proliferation at 5 μM, 10 μM, and 25 μM with 6 h of incubation. The application of 50 μM resveratrol markedly decreased the viability of cells (*p* < 0.05) (Figure 5A). Pretreatment of resveratrol (5 μM and 10 μM) for 6 h significantly attenuated the decreased cell viability induced by 400 μM H_2_O_2_ for 6 h (*p* < 0.05) (Figure 5B). Based on these results, subsequent experiments were conducted using 5 μM resveratrol.

### 3.5. Effect of Resveratrol on the Antioxidant Ability and Apoptosis of IDECs Exposed to H_2_O_2_

**Figure 6:**
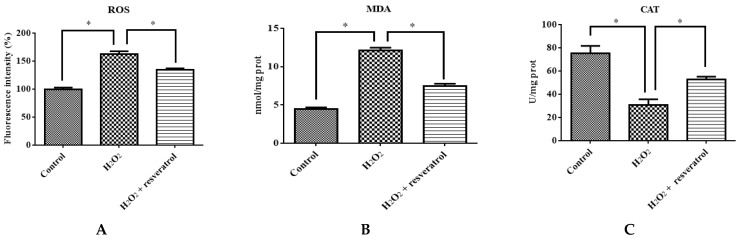

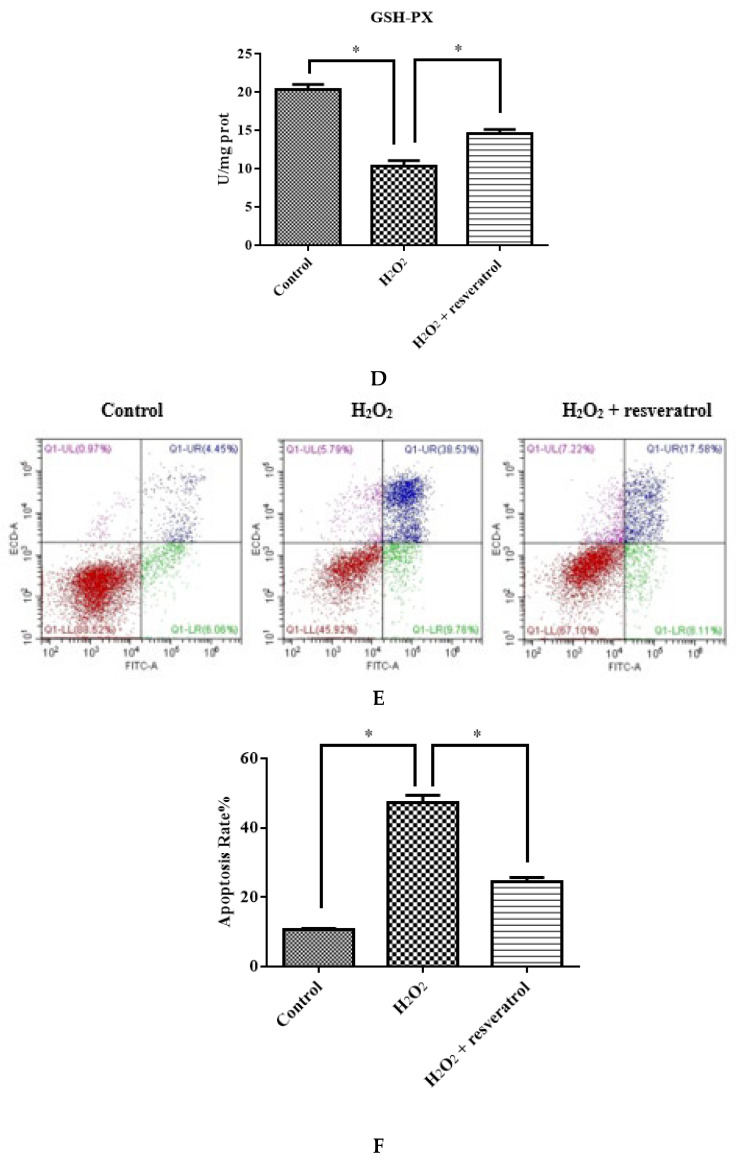
IDECs were pretreated with 5 µM resveratrol and then cocultured with 400 μM H_2_O_2_ for 6 h. (**A**) The ROS production in IDECs treated with different groups was determined by 2′,7′-dichlorofluorescein diacetate (DCFH-DA). (**B**) The activities of MDA in IDECs were detected by commercial kits. (**C**) The activities of CAT in IDECs were detected by commercial kits. (**D**) The activities of GSH-PX in IDECs were detected by commercial kits. (**E**) Apoptosis cells were analyzed by flow cytometry using Apoptosis Detection Kit. (**F**) The data were analyzed through one-way ANOVA and are expressed as the mean ± SE (*n* = 3). * *p* < 0.05 compared to H_2_O_2_ group.

To confirm that resveratrol protects against the H_2_O_2_-induced oxidative stress damage of IDECs, ROS, MDA, CAT and GSH-PX levels were determined in 400 μM H_2_O_2_-treated IDECs pretreated with 5 μM resveratrol. Pretreatment with resveratrol for 6 h reduced ROS production in IDECs under oxidative stress (*p* < 0.05) (Figure 6A). The addition of 5 μM resveratrol significantly reduced the MDA level and markedly increased the CAT level compared with the H_2_O_2_-induced group (Figure 6B–D).

The total apoptosis rate of different treatment groups was detected to determine whether the protective effect of resveratrol was associated with apoptosis reduction. The results indicated that the resveratrol treatment significantly decreased the total apoptosis rate (*p* < 0.05) (Figure 6E,F).

### 3.6. Resveratrol Increased the Expression of p-AKT, p-P38, NRF2, and p-NRF2 Proteins in H_2_O_2_-Induced IDECs

**Figure 7:**
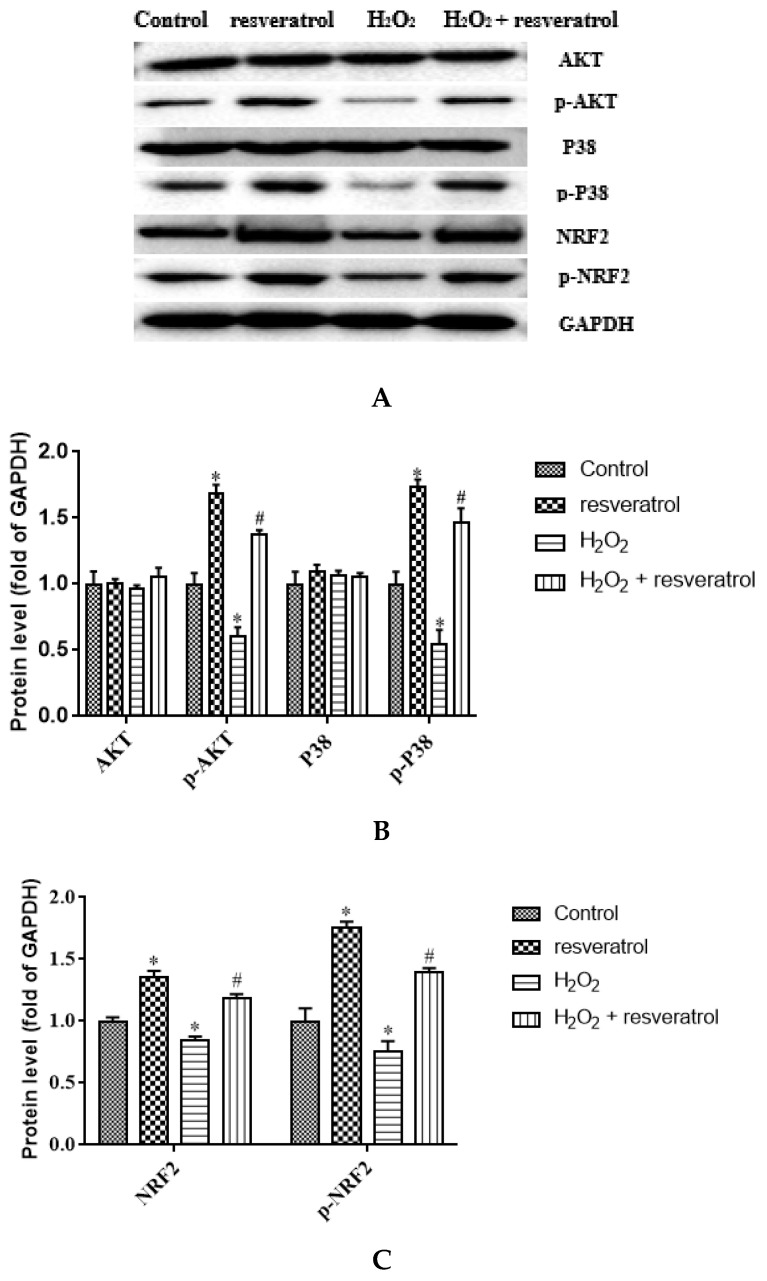

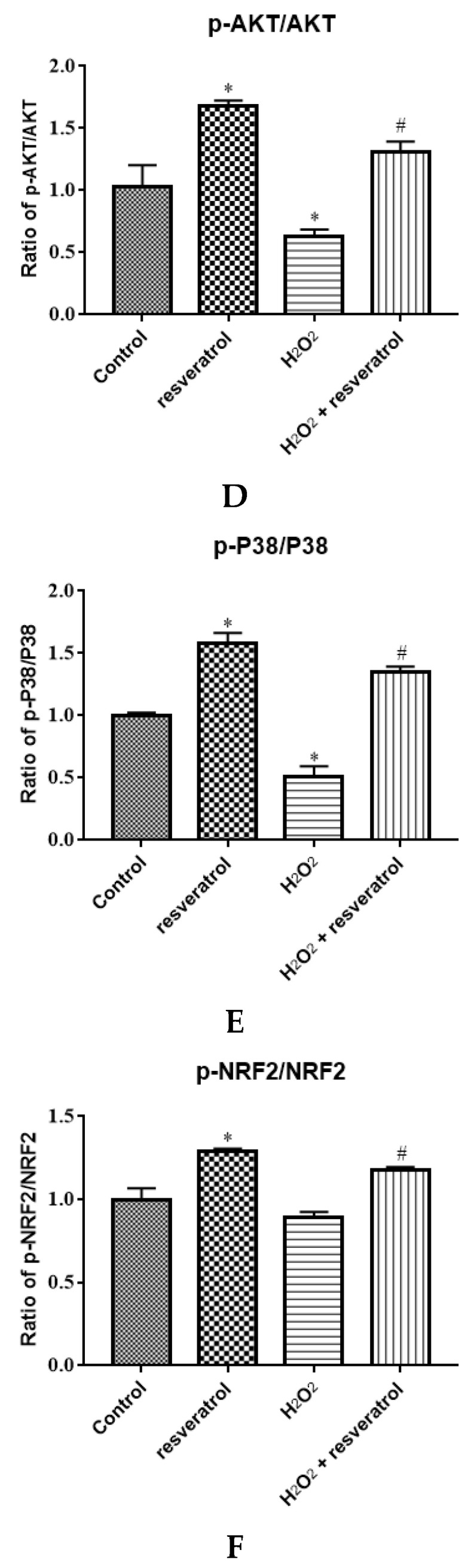
IDECs were pretreated with 5 µM resveratrol for 6 h and then cocultured with 400 μM H_2_O_2_ for 6 h. (**A**) Protein levels of AKT, p-AKT, P38, p-P38, NRF2, p-NRF2, and GAPDH were detected by Western blot. (**B**) The expression of AKT, p-AKT, P38, and p-P38 was quantified by densitometry, and data were normalized to GAPDH. (**C**) The expression of NRF2 and p-NRF2 was quantified, and data were normalized to GAPDH. (**D**) Histogram of p-AKT/AKT in different treated IDECs. (**E**) Histogram of p-P38/P38 in different treated IDECs. (**F**) Histogram of p-NRF2/NRF2 in different treated IDECs. The data were analyzed through one-way ANOVA and are expressed as the mean ± SE (*n* = 3). * *p* < 0.05 compared to control and ^#^ *p* < 0.05 compared to H_2_O_2_ group.

Given that AKT and P38 are key proteins involved in important cellular cascades mediating cell survival and NRF2 is the central regulator of the oxidative stress response, the expression of related proteins was measured. The levels of p-AKT and p-P38 were significantly decreased in the H_2_O_2_-treated group compared with the control group (*p* < 0.05) (Figure 7A,B), whereas resveratrol treatment reversed the H_2_O_2_-induced down-regulation of p-AKT and p-P38 (*p* < 0.05). There were no differences in the levels of AKT and P38 among the groups. The expression of NRF2 and p-NRF2 decreased significantly in the H_2_O_2_-treated group compared with the control group (*p* < 0.05), whereas the levels of NRF2 and p-NRF2 were markedly increased by resveratrol (*p* < 0.05) (Figure 7C). The p-AKT/AKT ratio and the p-P38/P38 were significantly lower in H_2_O_2_-induced IDECs than in control cells (*p* < 0.05), and the addition of resveratrol was able to reverse this pattern (*p* < 0.05) (Figure 7D,E).The p-NRF2/NRF2 ratio has no significant difference between in the H_2_O_2_-treated group and in the control group (*p* > 0.05), but the p-NRF2/NRF2 ratio was obviously increased by resveratrol (*p* < 0.05) (Figure 7F).

### 3.7. Resveratrol Regulated the Expression Levels of Apoptosis-Related Proteins in H_2_O_2_-Induced IDECs

**Figure 8:**
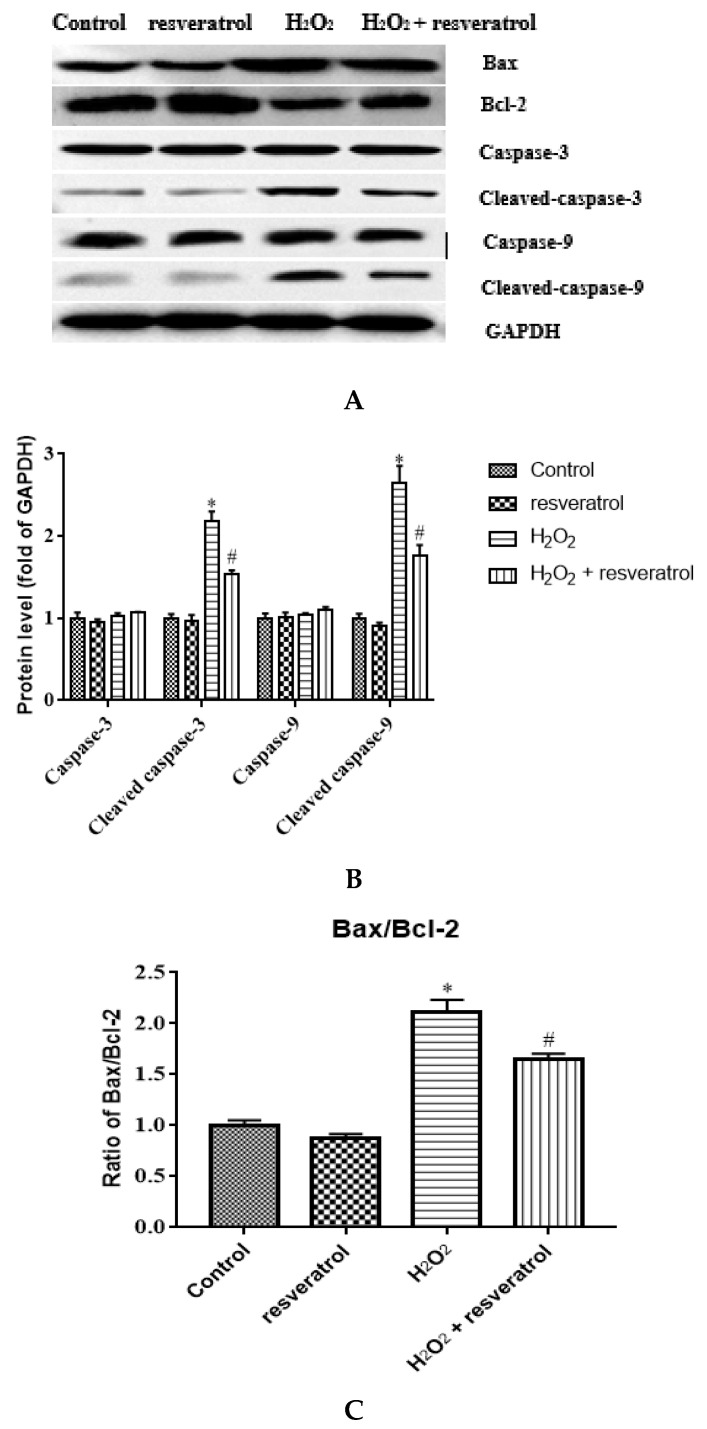
IDECs were pretreated with 5 µM resveratrol for 6 h and then cocultured with 400 μM H_2_O_2_ for 6 h. (**A**) Protein levels of Bax, Bcl-2, caspase-3, cleaved caspase-3, caspase-9, and cleaved caspase-9 were detected by Western blot. (**B**) The expression of caspase-3, cleaved caspase-3, caspase-9 and cleaved caspase-9 was quantified by densitometry, and data were normalized to GAPDH. (**C**) Histogram of Bax/Bcl-2 in different treated IDECs. The data were analyzed through one-way ANOVA and are expressed as the mean ± SE (*n* = 3). * *p* < 0.05 compared to control and ^#^ *p* < 0.05 compared to H_2_O_2_ group.

To clarify the role of resveratrol in the apoptosis of IDECs, the expression levels of apoptosis-related proteins such as Bax, Bcl-2, caspase-3, cleaved caspase-3, caspase-9, and cleaved caspase-9 were detected by Western blot. There was a marked increase in the expression levels of cleaved caspase-3 and cleaved caspase-9 after H_2_O_2_ treatment compared with the control group (*p* < 0.05), whereas resveratrol treatment significantly inhibited the increase in the levels of these two proteins (*p* < 0.05) (Figure 8A-B). The Bax/Bcl-2 ratio was significantly higher in H_2_O_2_-induced IDECs than in control cells (*p* < 0.05), and the addition of resveratrol was able to reverse this pattern (*p* < 0.05) (Figure 8C).

## 4. Discussion

The intestine is a major digestive and absorptive organ regulating the intake of nutrients that provides a barrier against macromolecules and pathogenic microorganisms. Oxidative stress can disrupt the intestinal barrier [24,25]. Therefore, the identification of natural substances that can counteract oxidative stress is a major focus of current research. In vitro cell models can be used to explore the protective and toxic effects of antioxidants, and the results obtained from in vitro experiments are often more intuitive compared with in vivo experiments [26]. The functions of feed additives on intestinal epithelial cells can only be tested in ducks in vivo because an in vitro cell culture system is currently lacking. In this study, we isolated and cultured primary duck intestinal epithelial cells from the small intestine of duck embryos following the methods of a previous study [22]. Primary duck intestinal epithelial cells possessed a homogeneous cobblestone-like morphology, which is similar to the growth of small intestine epithelial cells in other animals [27,28].

*SV40T* is currently the most widely used gene for inducing cell immortalization; it abolishes the inhibition of the cell cycle by inactivating p53 and pRB [29]. A large number of *SV40T*-immortalized cells have been constructed with biological characteristics comparable to primary cells. Recently, *SV40T* has been used to establish an immortalized rabbit melanocyte cell line [30], mice epicardial cell line [31], and sheep embryo kidney cell line [5]. According to these previous studies, we hypothesize that *SV40T* could be used to obtain IDECs. The results of qRT-PCR experiments revealed that *SV40T* mRNA was present in IDECs but not in primary cells. Cytokeratins play an important role in maintaining the overall structural integrity of epithelial cells. CK18, a member of the keratin family, is considered a marker of epithelial cells [3,4,22]. In this study, the expression of CK18 was positive in primary intestinal epithelial cells and IDECs, demonstrating that the cells were epithelial in origin. IDECs were able to be passaged in vitro for more than 30 generations owing to the successful transfection of *SV40T*. Therefore, we conclude that the newly established cell line (IDECs) could be used to conduct research on oxidative stress or other intestinal diseases in ducks.

The cytotoxicity of H_2_O_2_ is related to oxidative stress and is mainly characterized by an increase in reactive oxygen and the disruption of antioxidant defense, which leads to a decrease in cell viability [13,32]. According to our results, H_2_O_2_ affected cell viability in a time-dependent and dose-dependent manner, which is consistent with the results of previous studies [21,33,34]. Oxidative stress is caused by the penetration of the oxidants produced in the cell membrane and the production of ROS, which leads to changes in the level of MDA, CAT, and GSH-PX [33,35]. The oxidative stress induced by ROS is an important mediator of apoptosis in epithelial cells [13]. Our results indicated that the level of ROS and the activity of MDA and CAT participating in antioxidant defense in IDECs were significantly altered under H_2_O_2_ treatment.

Resveratrol, which is a polyphenolic compound contained in various fruits and herbs, has antioxidant and anti-inflammatory functions [36,37,38]. However, a high concentration of resveratrol significantly inhibits cell proliferation. Treatment with 100 nM resveratrol was suitable for exploring its protective effect on human umbilical vein endothelial cells [39]. Cells markedly differ in their responses to antioxidant substances because of differences in physical conditions [22]. Resveratrol has been reported to up-regulate the activity of CAT, SOD and GSH-PX and maintain intracellular ROS homeostasis in IPEC-J2 cells under H_2_O_2_-induced oxidative stress [18]. Wang et al. showed that resveratrol could protect Caco-2 cells against H_2_O_2_-induced oxidative stress by reducing the levels of MDA and ROS [40]. Besides, resveratrol enhanced the duck serum antioxidant capacity by decreasing the level of MDA and increased the CAT activity in the jejunum of ducks under heat stress [41]. Yang et al. found that dietary resveratrol alleviates intestinal epithelial dysfunction in LPS-induced duck ileitis [42]. The results of a previous study have shown that resveratrol protects against the oxidative stress of retinal pigment epithelium cells by modulating MDA activity [43].

The transcription factor Nrf2 is an important sensor of oxidative stress that regulates antioxidant and phase 2 detoxifying enzymes and related proteins in cells [44]. Several studies have demonstrated that resveratrol treatment dramatically up-regulates the expression of Nrf2 in vivo and in vitro [45,46,47]. Under oxidative stress conditions, the phosphorylated Nrf2 (p-Nrf2 (Ser40)) is formed from Nrf2 and transferred into the nucleus to encode conserved antioxidant enzymes [48]. The PI3K/AKT and P38 MAPK signal pathways are involved in Nrf2-dependent transcription in diverse cell types in response to ROS injury. Previous experiments have shown that the level of phosphorylated P38 decreases in mouse liver sinusoidal endothelial cells under oxidative stress [49], and chlorogenic acid protects MC3T3-E3 cells against oxidative stress through the PI3K/Akt-mediated Nrf2 signal pathway [50]. Phosphorylation is an activated form of AKT protein and P38 protein, and phosphorylated AKT and P38 MAPK participate in the regulation of oxidative stress [51,52]. In our study, resveratrol up-regulated the levels of NRF2, p-NRF2, p-AKT, and p-P38 in IDECs under oxidative stress, suggesting that resveratrol attenuates oxidative stress by activating related signal pathways.

Apoptosis, which can be triggered by various factors outside or inside cells, can be induced by oxidative stress [53]. Excessive apoptosis initiated by extracellular agents is considered a pathologic lesion. In this study, H_2_O_2_ significantly induced the apoptosis of IDECs; however, resveratrol treatment significantly decreased the apoptosis rate in IDECs exposed to H_2_O_2_. This result indicated that resveratrol can protect IDECs against H_2_O_2_-induced apoptosis. Bax and Bcl-2, which belong to the Bcl-2 protein family, are involved in the regulation of cell apoptosis, and caspases play an important role during the initiation and effector phases of apoptotic cell death; the ratio of Bax to Bcl-2 is used to evaluate the rate of apoptosis, as a higher ratio of Bax to Bcl-2 is associated with a higher rate of apoptosis [54]. Caspase-3 and caspase-9 are potential effectors of apoptosis that are triggered via several different pathways [55]. Previous studies have demonstrated that H_2_O_2_ regulates the balance between Bcl-2 and Bax and activates caspase cascades that subsequently lead to apoptosis [56,57]. Our results showed that resveratrol significantly decreased the levels of cleaved caspase-3 and cleaved caspase-9 compared with H_2_O_2_-treated IDECs, and the ratio of Bax to Bcl-2 was lower in IDECs under oxidative stress after resveratrol treatment. These findings suggest that resveratrol inhibits cell apoptosis by up-regulating cleaved caspase-3 and cleaved caspase-9 expression and altering the ratio of Bax to Bcl-2.

## 5. Conclusions

The newly established IDECs in this study retain the morphological and functional features of primary duck intestinal epithelial cells, suggesting that they provide a robust tool for the in vitro study of duck epithelial cells. Subsequently, we constructed an oxidative stress cell model using H_2_O_2_. Our results indicated that resveratrol might alleviate the oxidative stress of IDECs by the PI3K/AKT and P38 MAPK signal pathways and inhibit apoptosis by altering the levels of cleaved caspase-3, cleaved caspase-9, Bax, and Bcl-2.

## Figures and Tables

**Table 1:** Characteristics of the primers used for the real-time PCR analysis.

Genes	Primer (from 5′ to 3′)	Accession Number
*SV40T*	F: ACTGAGGGGCCTGAAATGA	M99358.1
	R: GACTCAGGGCATGAAACAGG	
*GAPDH*	F: CTTTGGACGCTGCTGTTG	XM_005016745
	R: GCTGTCACCGTTGAAGTCG	

**Table 2:** Quantitative reverse transcription polymerase chain reaction threshold (Ct) values.

Gene	Primary Duck Intestinal Epithelial Cells	Immortal Duck Intestinal Epithelial Cells
*GAPDH*	19.72 ± 0.07	19.83 ± 0.03
*SV40T*	35.57 ± 0.17 ^a^	24.92 ± 0.16 ^b^

Values are given as mean ± SE of three independent experiments. Mean values sharing different superscripts within the same row differ significantly (*p* < 0.05).

## Data Availability

Data is contained within the article.

## Abbreviations

IDECs	Immortalized duck intestinal epithelial cells
*SV40T*	Simian virus 40 Large T
CK18	Cytokeratin 18
*hTERT*	Human telomerase reverse transcriptase
ROS	Reactive oxygen species
MDA	Malondialdehyde
CAT	Catalase
SOD	Superoxide dismutase
NRF2	Nuclear factor erythroid 2-related factor 2
GSH-PX	Glutathione peroxidase
